# An interview with Ifrah Zawar, 2023 *Epilepsia Open* prize winner for clinical research

**DOI:** 10.1002/epi4.12763

**Published:** 2023-07-05

**Authors:** Aristea S. Galanopoulou

**Affiliations:** ^1^ Neurology and Neuroscience Albert Einstein College of Medicine New York New York USA

**Figure:**
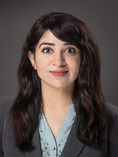
Ifrah Zawar

## 1 PLEASE TELL US ABOUT YOURSELF

I was born and raised in Pakistan. I received my medical degree from Aga Khan University (Karachi, Pakistan). In 2014, after medical school, I moved to the United States to pursue my neurology residency and then epilepsy fellowship at the Cleveland Clinic in Ohio. At the Cleveland Clinic, I also served as Chief Epilepsy Fellow. After completing my training, I joined the University of Virginia (UVA) in the fall of 2021, where I am currently an Assistant Professor of Neurology, NIH NeuroNEXT fellow, and a graduate student working toward my Master's degree in clinical research.

**FIGURE 1:**
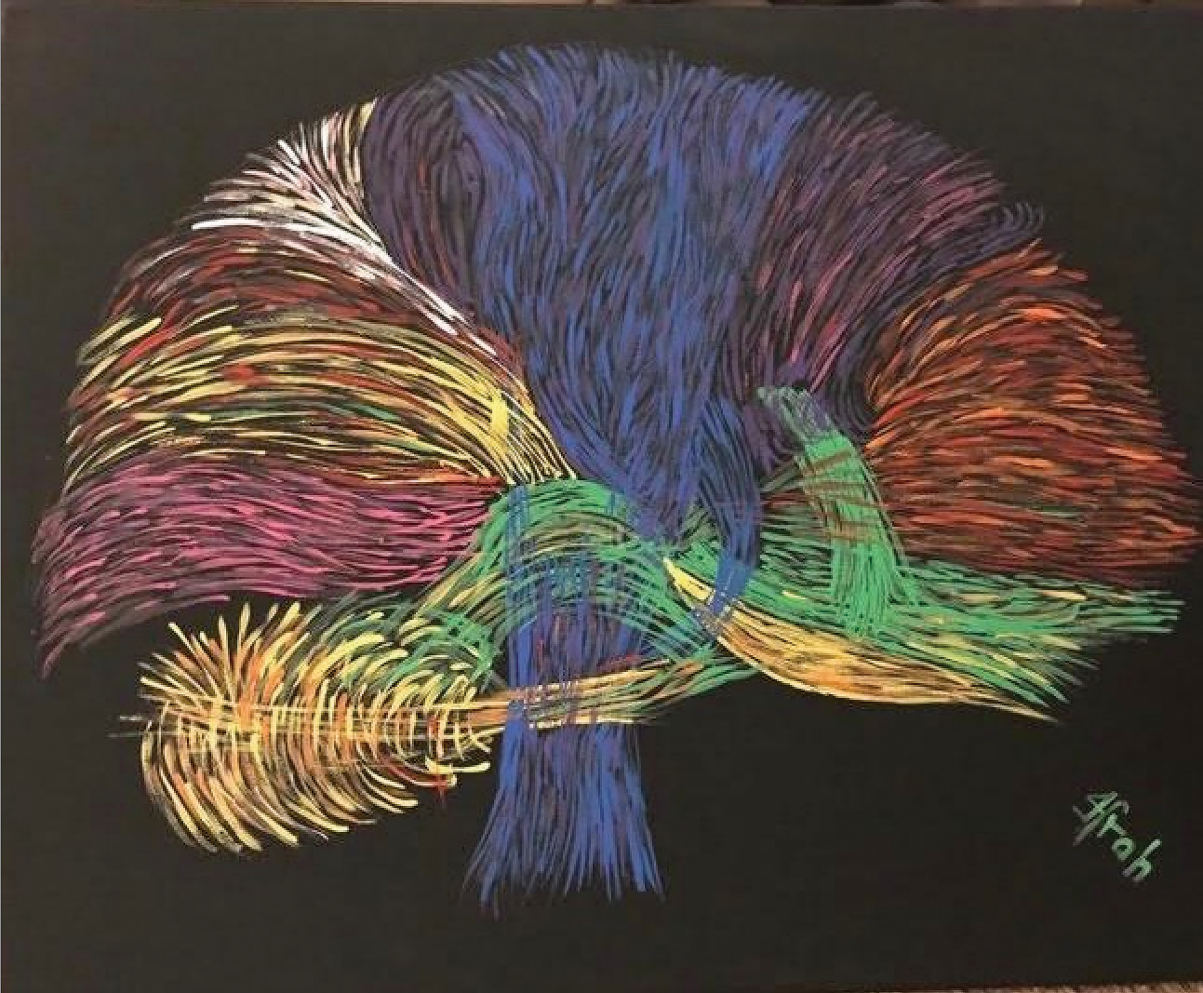
“Hues of the brain”, a painting by Dr Ifrah Zawar.

In my spare time, I enjoy spending time with my family and friends (Figure 1), paint (brains and EEG), and travel.

## 2 HOW DID YOU BECOME INTERESTED IN CONDUCTING RESEARCH IN THIS FIELD?

During my neurology residency, no other subspecialty captivated me like epilepsy. In epilepsy, I have learned to truly admire the beauty of functional neuroanatomy. It fascinates me how seizure semiology can help localize epileptic zones and can so accurately pinpoint a particular area in the brain, how EEG can detect subtle neuronal dysfunction in the brain that even the most advanced imaging studies cannot detect, how seizure control can totally change a patient's life, and how adequate seizure control can largely prevent sudden unexpected death in epilepsy (SUDEP) and other co‐morbidities associated with epilepsy. I recognized in my residency that epilepsy is one of the most debilitating neurologic diseases, not only for the patients but a struggle for their entire families. As I saw epilepsy in its best form and its worst, it became a lifelong passion. Having worked with some of the most renowned epilepsy physicians at the Cleveland Clinic further inspired me to pursue my career in epilepsy.

I recognized the significance of research early on and decided that patient‐centered research would be an integral part of my career. I have been actively involved in clinical research in medical school, residency, fellowship, and now as a faculty. The more I have learned in this field, the more I have wanted to learn. Research in epilepsy is a promise to all its sufferers of a better life. There is an incredible amount that has yet to be discovered, and I wish to be part of that process for the rest of my life.

## 3 PLEASE EXPLAIN THE QUESTION YOUR STUDY ADDRESSED, AND HOW YOU DESIGNED IT

Timely seizure detection with continuous EEG (cEEG) has the potential to reduce in‐hospital mortality and complications. However, cEEG is a resource‐intensive tool. Therefore, it is important to identify patients at risk for delayed seizure detection who may benefit from prolonged cEEG monitoring. While coma is known to be associated with delayed seizure detection, several other factors have not been studied previously. The purpose of my research was to determine additional risk factors that predict delayed seizure detection and to determine the optimal duration of cEEG to exclude subclinical seizures in a large patient population. I identified 2402 adult patients who underwent cEEG at the Cleveland Clinic in 2016. Data were collected from electronic medical records and a review of cEEG records was performed to determine the time to detect subclinical seizures.

## 4 WHAT WERE THE RESULTS AND HOW DO YOU INTERPRET YOUR FINDINGS?

My study found that seizure detection increased linearly till 36 h of monitoring, and the odds of seizure detection increased by 46% for every additional day of monitoring. Considering the linear trend in seizure detection, standard monitoring of 36 h may be more effective than 24 h, particularly for high‐risk patients. For awake patients without interictal epileptiform discharges, <24 h of cEEG appeared optimal. We found several risk factors for delayed seizure detection, including stupor, lethargy, lateralized (LPDs) or generalized periodic discharges (GPDs), acute brain insults, brain hemorrhage, especially multiple concomitant bleeds, altered mental status as primary cEEG indication, and use of anti‐seizure medications at cEEG initiation. Longer cEEG (≥48 h) may need to be considered for these high‐risk patients.

These important findings can make significant future contributions to the management of hospitalized patients on cEEG. With the development of a standard duration of cEEG monitoring tailored according to patient clinical and EEG characteristics, my research findings may help manage patients better as well as increase the likelihood of detecting seizures in high‐risk patients.

## 5 WHAT ARE THE NEXT STEPS THAT YOU PLAN TO TAKE, AND WHAT ARE YOUR CAREER GOALS?

My background and experiences have contributed significantly to my growth as a physician and a researcher. Over the years, my passion for EEG and epilepsy research has continued to grow. As I have navigated through my research career, I recognized that despite the highest incidence of epilepsy in older adults and its associated co‐morbidity, this area remains understudied. It is here that I have identified my niche in epilepsy research. My long‐term goal is to become a leading clinician scientist in the field of epilepsy in older adults and to develop prevention and treatment strategies that will control seizures and improve cognitive, functional, and mortality outcomes for these patients.

Similar to my previous research, which identified the risk factors for delayed seizures on cEEG and the optimal duration of cEEG, my current research is focused on identifying the risk factors for seizures among older adults and those with cognitive impairment. My lab is also conducting a prospective study funded by Alzheimer's Association to study EEG and other biomarkers among patients with Alzheimer's disease.

## 6 WHAT DOES THE *EPILEPSIA OPEN* PRIZE MEAN FOR YOU, YOUR LABORATORY, RESEARCH INSTITUTE, AND YOUR FUTURE?

I am extremely grateful to receive such a prestigious award at such an early stage of my career. I am thankful to my research mentor, Dr. Hantus, for the conception of the project idea and for his guidance through this work as well as to several other mentors at the Cleveland Clinic and UVA and my family, who have always supported me. I am eternally grateful and truly in debt to my research mentors, colleagues, and my department chair, who have been immensely helpful in shaping my research career thus far and continue to help me every step of the way. This award is such a great honor for me and my lab and an acknowledgment that hard work always pays off. This prize has further strengthened my drive to continue meaningful clinical research in epilepsy.

Read the winning article “Risk factors that predict delayed seizure detection on continuous electroencephalogram (cEEG) in a large sample size of critically ill patients.”

